# Mobilizing Breast Cancer Prevention Research Through Smartphone Apps: A Systematic Review of the Literature

**DOI:** 10.3389/fpubh.2019.00298

**Published:** 2019-11-06

**Authors:** Lauren C. Houghton, Renata E. Howland, Jasmine A. McDonald

**Affiliations:** ^1^Mailman School of Public Health, Columbia University Irving Medical Center, New York, NY, United States; ^2^Herbert Irving Comprehensive Cancer Center, Columbia University Irving Medical Center, New York, NY, United States

**Keywords:** breast cancer, cancer control continuum, mobile application, smartphone, prevention, systematic review

## Abstract

**Background:** Breast cancer rates have been increasing worldwide, particularly among young women, suggesting important interactions between genes and health behaviors. At the same time, mobile technology, including smartphones applications (apps), has emerged as a new tool for delivering healthcare and health-related services. As of 2018, there were nearly 600 publicly available breast cancer apps designed to provide disease and treatment information, to manage disease, and to raise overall awareness. However, the extent to which apps are incorporated into breast cancer prevention research is unknown. Therefore, the objective of this review was to determine how mobile applications are being used for breast cancer prevention among women across the cancer control continuum.

**Methods:** Using the Preferred Reporting Items for Systematic Reviews and Meta-Analyses (PRISMA) guidelines, we searched PubMed and Web of Science Core Collection databases using the keywords breast cancer, smartphone, mobile application, and phone app. Full-length journal articles available in English that addressed the research question were included. We categorized articles by prevention type (primary, secondary, and tertiary) and phase of research (protocol, development, feasibility, pilot, measurement, and effectiveness), and identified common themes and gaps.

**Results:** Our search yielded 82 studies (69 unique) that used apps in breast cancer prevention research across 20 countries. Approximately half of the named apps were publicly available. The majority (73%) of studies targeted tertiary prevention; 15% targeted secondary and 13% targeted primary prevention. Apps were used across all phases of research with the predominant phase being feasibility in tertiary prevention (34%), effectiveness in secondary prevention (63%), and development (30%) and effectiveness (30%) in primary prevention. Common uses included assessing outcomes relevant to clinical care coordination, quality of life, increasing self-efficacy and screening behaviors, and tracking and managing health behaviors.

**Conclusions:** We identified the following gaps: few effectiveness studies in tertiary prevention, minimal use of apps for breast cancer etiology or early detection, and few interventions in those at average risk of breast cancer. These findings suggest that while mobile apps can inform breast cancer prevention across the continuum, more work is needed to incorporate apps into primary prevention.

## Introduction

Breast cancer rates have been increasing worldwide, particularly among young women (1). Such rapid changes in the incidence of early onset breast cancer cannot be attributed solely to genetics, but rather to interactions between health behaviors and genes. Given many behavioral risk factors for breast cancer are modifiable, public health prevention and intervention studies have long sought to change individual health behaviors and more recent work recognizes that a multi-faceted approach is needed to address these behaviors because they are complex in nature (2).

At the same time, mobile technologies, including smartphone applications (hereafter referred to as apps), have emerged as new tools for delivering healthcare and health-related services in the field of cancer and particularly breast cancer. In fact, nearly half of all cancer apps are targeted toward breast cancer (3). A recent review suggests there are nearly 600 publicly available breast cancer apps designed to provide disease and treatment information, to manage disease, and to raise overall awareness (4). With the widespread availability and use of applications, researchers have an opportunity to leverage this ubiquitous technology for breast cancer prevention. However, the extent to which apps are incorporated into breast cancer prevention research across the cancer control continuum is unknown.

Given that the use of apps for breast cancer prevention is still in the early stages of adoption, the authors agreed that a systematic review with a broad research scope was warranted. Therefore, we performed a systematic review to answer the question: how are mobile apps being used for breast cancer prevention research across the cancer control continuum, including tertiary, secondary, and primary prevention, in women? Since the use of apps in research is relatively new, we also sought to identify at what phases of the research process mobile apps were being used for breast cancer research, including protocol, development, feasibility, pilot, effectiveness, and measurement studies. In addition to the systematic review, we sought to find common themes and gaps across the body of literature.

## Methods

### Search Strategy

In order to conduct this systematic review, we utilized the Preferred Reporting Items for Systematic Reviews and Meta-Analyses (PRISMA) guidelines (5). We systematically reviewed PubMed and Web of Science Core Collection databases in December 2018 (updated February 7, 2019 to ensure the most recent articles were captured). Search terms included breast cancer, smartphone, mobile application, and phone app. These terms were applied to all fields in order to capture the greatest number of articles. We also employed the controlled vocabulary of Medical Subject Headings (MeSH), available in PubMed only, including subheadings, for breast neoplasms and mobile apps. Supplementary Table 1 includes the complete search string as it was conducted in PubMed. We searched for additional articles using the terms mHealth, health app, breast cancer app, iPhone application, and Android application. Our search contained no restrictions regarding language or year of publication. All references were exported to Endnote (X8, Thompson Reuters). We first removed duplicate citations using the automatic feature and then manually reviewed articles for additions that had minor differences in the way information was indexed.

### Inclusion/Exclusion Criteria

Records were screened in Endnote and included if they were published as an original research article in English. The primary reviewer [RH] then reviewed the full-text article for relevance to the study question. Articles were excluded if study participants were providers or caregivers; if breast cancer prevention was not an explicit goal or implication of the research; if the article did not include a mobile application or only discussed that the research could be potentially adapted into a mobile application; or if the smartphone was examined as a carcinogen. We also excluded books or book chapters, meeting abstracts, non-empirical records (e.g., reviews, editorials, and letters), non-English records, and records where the full-text were unavailable. When inclusion was unclear, authors LH and JAM independently reviewed the articles and then all authors discussed until a consensus was met. LH and JAM also reviewed 20% of excluded articles for accuracy. In one case where we could not reach consensus, we contacted the corresponding author for clarification. Among all studies that were eligible for qualitative analysis (*n* = 82), we flagged those studies that had multiple publications reporting outcomes across different stages of research (e.g., a protocol and effectiveness study) but were using the same underlying cohort (*n* = 23).

### Data Extraction and Analysis

For studies meeting the inclusion criteria, the primary reviewer [RH] extracted the following information from eligible studies: population characteristics, sample size, location of the study (country), mobile application name (where applicable), and study objectives and/or outcomes (e.g., quality of life, efficacy, literacy). We categorized studies by prevention type based on whether they were targeting a secondary cancer event and/or morbidity/mortality (tertiary), early diagnosis and treatment (secondary), or disease prevention (primary). We assigned articles to only one prevention type category. We also categorized studies by research phase based on the study outcome(s). Studies categorized as Development included those collecting information on participant interest and preferences for a mobile application that was not yet produced. Based on features outlined by Orsmond and Cohn (6), we categorized Feasibility studies as those that reported process outcomes, such as usability of an app (6). We categorized Pilot studies as those studies where the author(s) self-described the study as such and/or the authors(s) mention that a larger study was being planned to evaluate the effectiveness of an intervention. Generally, Pilot studies reported outcomes among a small sample, where the average sample size was ~35. Effectiveness studies reported outcome measures from a full study; and a Protocol described the protocol for a study, such as for an effectiveness study, usually in the title of the article itself. Measurement studies were those that reported outcomes related to validity or reliability. Some studies were categorized across multiple research phases if papers combined multiple outcomes; therefore, research phase categories were not mutually exclusive.

Our initial analysis tabulated all articles eligible for qualitative analysis by cancer prevention type and by research phase. We then estimated the number of articles published by year. We used the subset of unique studies and tabulated the number of publications by country and continent. Lastly, void of *a priori* hypotheses regarding common themes and gaps in the literature, we comprehensively reviewed unique studies by cancer prevention type to identify common themes and gaps. We then extracted mobile app details and categorized app use by prevention type and the availability of the app in the Apple and/or Android app store.

## Results

**Figure 1:**
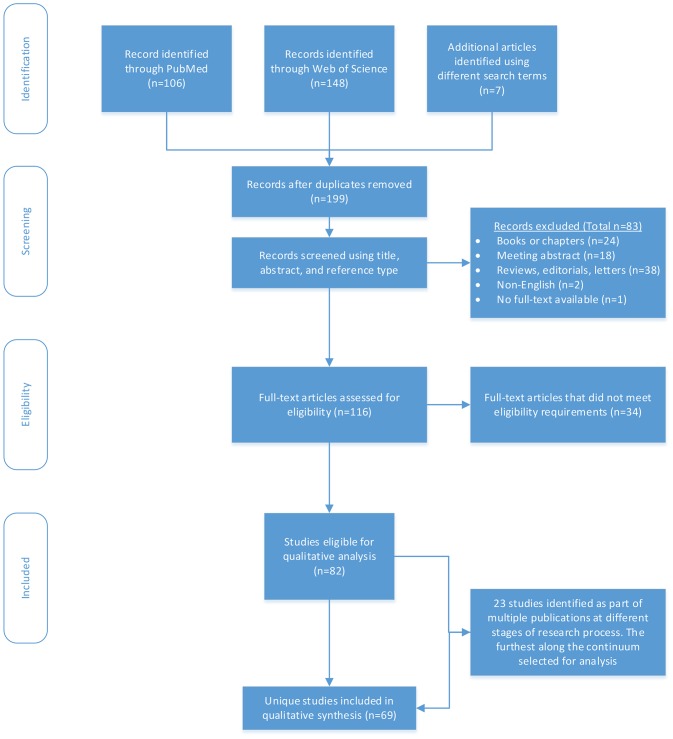
Flow chart of systematic review.

We identified 199 records through our search, excluding duplicate records (Figure 1). Of these, we first screened the record title, abstract, and reference type for eligibility and excluded 83 records as ineligible. We then assessed the remaining 116 articles for eligibility through full-text review and further excluded 34 records. We identified 82 studies eligible for qualitative analysis. Of the 82, we identified 23 studies that were part of multiple publications that used the same underlying cohort to report outcomes across different research phases. Therefore, we identified 69 unique studies, 75% (*n* = 52) were tertiary, 12% (*n* = 8) were secondary, and 13% (*n* = 9) were primary.

### The Use of Mobile Apps by Cancer Prevention Type and Research Phase

**Figure 2:**
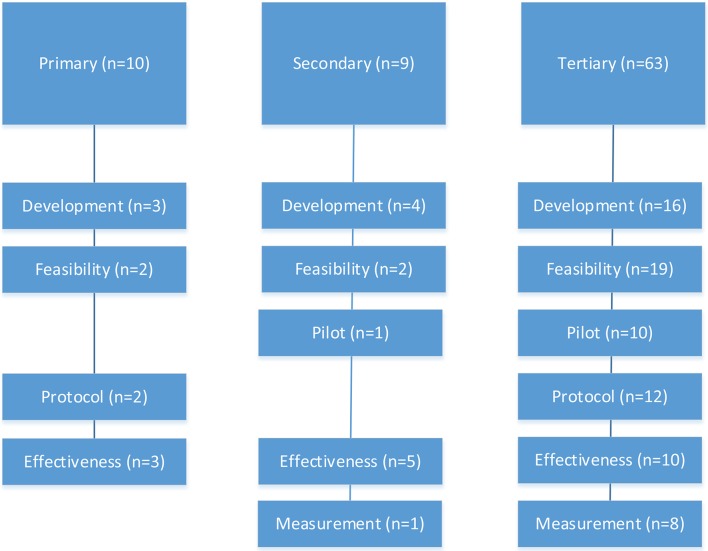
The use of mobile apps across primary, secondary, and tertiary breast cancer prevention by research phase (*n* = 82 eligible studies).

As displayed in Figure 2, apps were used across all phases of research with the predominant phase being feasibility in tertiary prevention studies (34%), effectiveness in secondary prevention studies (63%), and development (30%) and effectiveness (30%) in primary prevention studies. Across the cancer prevention continuum, 14 studies were protocols (17%), 23 were development (28%), 23 were feasibility (28%), 11 were pilots (13%), 18 were effectiveness (22%), and 9 were measurement studies (11%). Given 23 articles reported on multiple study phases, the categories were not mutually exclusive and percentages exceed 100%.

### Mobile App Use: Growth and Global Reach

**Figure 3:**
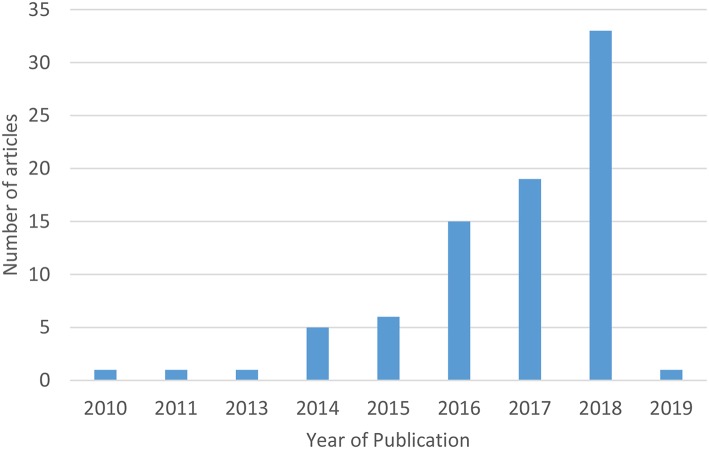
Number of studies using mobile apps for breast cancer prevention research among women by year of publication (*n* = 82 eligible studies). *The initial search was conducted in December 2018 and updated February 7, 2019.

**Figure 4:**
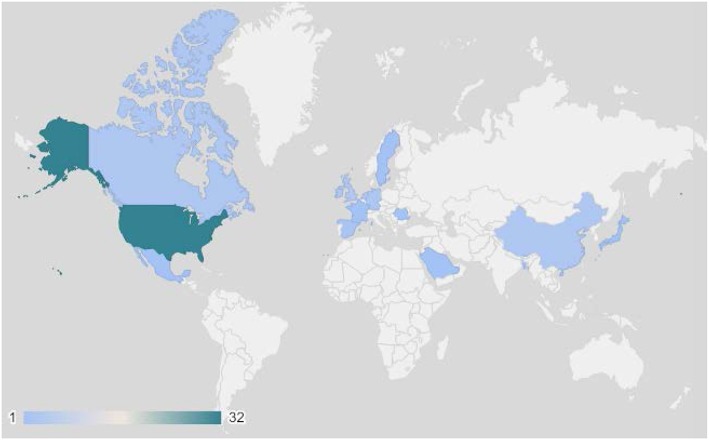
Number of publications by country (*n* = 69 unique studies).

The number of studies using apps for breast cancer prevention research increased rapidly over the last 10 years (Figure 3). The earliest studies in this review were published in 2010, while the majority (40%) were published in 2018. There was international use of apps in breast cancer prevention research, with the exception of Africa and South America (Figure 4). The studies included in this review were conducted in 20 countries, with most studies conducted in the US (43%) and more than one study each occurring in Canada (7–9), China (10–12), Germany (13–15), Ireland (16–18), Korea (19–24), the Netherlands (25–29), Spain (30, 31), and the United Kingdom (32–35). Tertiary prevention studies took place in North America (US, Canada, Mexico), Western Europe (UK, Sweden, Netherlands, Germany, France, Spain Ireland), and Asia (Korea, China, Japan, Singapore). Secondary prevention studies were based in North America (US), Asia (Korea, China, India, Bangladesh), and Eastern Europe (Romania). Primary prevention studies were based in North America (US), Europe (Netherlands), and the Middle East (Kingdom of Saudi Arabia).

### Review of Mobile Apps by Cancer Prevention Types: Common Themes

#### Tertiary Prevention

The majority of mobile apps used for breast cancer prevention research addressed tertiary prevention. We identified 63 studies (53 unique) (Table 1) and the articles ranged across research phases including development (24.5%), feasibility with a focus on process (34%), pilots with a focus on outcomes (18.9%), protocols (15.1%), effectiveness (16%), and measurement (11.3%) (Figure 2).

**Table 1:** Articles using mobile apps for tertiary breast cancer prevention (*n* = 63 eligible studies).

**References**	**Type of study**	**Population (sample size)**	**Location**	**Outcomes**
Ainsworth et al. (36)	Feasibility	Breast cancer survivors (40)	US	App use and experience
Akechi et al. (37)	Protocol	Breast cancer survivors (444)	Japan	Fear of recurrence
Ali et al. (38)	Development	Patients undergoing treatment for cancer (423)	Singapore	App interest and preferences
Armstrong et al. (8)	Effectiveness	Women undergoing breast reconstruction (65)	Canada	Post-surgical follow-up
Armstrong et al. (39)^*^	Protocol	Women undergoing breast reconstruction (72)	Canada	Post-surgical follow-up
Banas et al. (40)	Development	Breast cancer survivors, Hispanic (31)	US	App interest and preferences
Baseman et al. (41)	Feasibility	Breast cancer survivors and providers (11)	US	App interest and preferences
Brett et al. (34)	Development	Women undergoing treatment for breast cancer (20)	UK	App use and experience
Buscemi et al. (42)	Feasibility + Pilot	Breast cancer survivors, Hispanic (25)	US	App use and experience, Quality of life
Iacobelli et al. (43)^*^	Development	Breast cancer survivors, Hispanic (9)	US	App interest and preferences
Yanez et al. (44)^*^	Protocol	Breast cancer survivors, Hispanic (80)	US	Quality of life
Chalela et al. (45)	Protocol	Women undergoing treatment for breast cancer (120)	US	Medication adherence
Delrieu et al. (46)	Protocol	Women undergoing treatment for breast cancer (60)	France	Physical activity, app use
Douma et al. (28)	Feasibility + Measurement	Women undergoing treatment for breast cancer (72)	Netherlands	Physical activity, app use
Drewes et al. (13)	Development	Women undergoing treatment for breast cancer and physicians (168)	Germany	App interest and preferences
Egbring et al. (14)	Effectiveness	Women undergoing treatment for breast cancer (139)	Germany	Daily functional activity
El Shafie et al. (15)	Development	Patients undergoing treatment for cancer (breast or prostate) (200)	Germany	App interest and preferences
Foley et al. (17)	Pilot	Women undergoing treatment for breast cancer (39)	Ireland	Mental health
Gehrke et al. (47)	Development + Feasibility	Breast cancer survivors (11) and their nurses (3)	US	App interest and preferences
Harder et al. (33)	Development + Feasibility	Women undergoing treatment for breast cancer (9)	UK	App interest and preferences
Hwang (7)	Effectiveness	Women undergoing treatment for breast cancer (72)	Canada	Readmission, app use and experience
Kim et al. (23)	Effectiveness	Women undergoing treatment for breast cancer (76)	Korea	Medication adherence
Kim et al. (21)	Measurement	Women undergoing treatment for breast cancer (78)	Korea	Reliability
Klasnja et al. (48)	Effectiveness	Women undergoing treatment for breast cancer (9)	US	Self-management
Klasnja et al. (49)^*^	Development	Women undergoing treatment for breast cancer (3)	US	App interest and preferences
Kubo et al. (50)	Feasibility + Pilot	Patients undergoing treatment for cancer (28) and their caregivers (14)	US	App use and experience, distress and quality of life
Langer et al. (51)	Measurement	Women undergoing treatment for breast cancer and their partners (107 couples)	US	Relationship satisfaction
Langius-Eklof et al. (52)	Protocol	Patients undergoing treatment for cancer (150)	Sweden	Symptom distress
Lloyd et al. (53)	Development	Breast cancer survivors (279)	US	App interest and preferences
Lozano-Lozano et al. (30)	Protocol	Breast cancer survivors (80)	Spain	Quality of life
Lozano-Lozano et al. (54)^*^	Measurement	Breast cancer survivors (20)	US	Validity and test-retest reliability
Lyons et al. (55)	Protocol	Breast cancer survivors (120)	US	Physical activity
McCarroll et al. (56)	Pilot	Breast and endometrial cancer survivors (50)	US	Physical activity
Min et al. (20)	Feasibility	Women undergoing treatment for breast cancer (30)	Korea	App use and experience
O'Brien et al. (16)	Development	Breast clinic sample (200)	Ireland	App use and experience
Ormel et al. (29)	Feasibility + Pilot	Patient undergoing treatment for cancer or cancer survivors (32)	Netherlands	Physical activity, use and experience
Paredes-Aracil et al. (57)	Measurement	Breast cancer survivors (272)	Spain	Model validation and calibration
Paredes-Aracil et al. (31)^*^	Measurement	Breast cancer survivors (287)	Spain	Model validation and calibration
Park et al. (24)	Effectiveness	Women undergoing treatment for breast cancer (356)	Korea	Physical activity
Lee et al. (58)^*^	Feasibility	Breast cancer survivors (88)	Korea	App use and experience
Phillips et al. (59)	Protocol	Breast cancer survivors (256)	US	Physical activity, use and experience
Phillips et al. (59)	Feasibility	Breast cancer survivors (279)	US	App interest and preferences
Pope et al. (60)	Feasibility + Pilot	Breast cancer survivors (10)	US	Physical activity, use and experience
Quintiliani et al. (61)	Feasibility + Pilot	Breast cancer survivors (10)	US	App use and experience, weight management
Raghunathan et al. (62)	Development	Patients undergoing cancer treatment (631)	US	App interest and preferences
Ritvo et al. (9)	Protocol	Breast cancer survivors (107)	Canada	Physical activity, use and experience
Roberts et al. (35)	Development	Cancer survivors (breast, prostate, colorectal) (32)	UK	App interest and preferences
Rosen et al. (63)	Feasibility + Effectiveness	Breast cancer survivors (112)	US	Quality of life, use and experience
Smith et al. (64)	Development	Breast cancer survivors, African American (96)	US	App interest and preferences
Soto-Perez-De-Celis et al. (65)	Pilot + Feasibility	Patients undergoing cancer treatment (40)	Mexico	Physical activity, use and experience
Stubbins et al. (66)	Effectiveness	Breast cancer survivors (33)	US	Weight management
Timmerman et al. (25)	Measurement	Cancer survivors (18)	Netherlands	Physical activity, reliability
Uhm et al. (22)	Effectiveness	Breast cancer survivors (356)	Korea	Physical activity
Valle et al. (67)	Feasibility + Pilot	Breast cancer survivors, African American (35)	US	Weight management and physical activity
Walker et al. (68)	Development	Breast cancer survivors and nurses (12)	US	App use and experience
Weaver et al. (32)	Pilot	Patients undergoing treatment for cancer (breast or colorectal) (26)	UK	Medication use and perceived support
Xiaosheng et al. (11)	Protocol	Breast cancer survivors (60)	China	Quality of life
Young-Afat et al. (27)	Feasibility	Women undergoing treatment for breast cancer (15)	Netherlands	App use and experience
Zhang et al. (69)	Feasibility	Cancer survivors and workshop attendees (~150)	Europe	App use and experience
Zhu et al. (70)	Effectiveness	Women undergoing treatment for breast cancer (114)	China	Self-efficacy
Zhu et al. (12)^*^	Feasibility	Women undergoing treatment for breast cancer (13)	China	App use and experience
Zhu et al. (71)^*^	Protocol	Women undergoing treatment for breast cancer (108)	China	Self-efficacy
Zhu et al. (71)^*^	Development	Women undergoing treatment for breast cancer (114)	China	Quality of life

* *Duplicate articles are indented*. *US, United States; UK, United Kingdom*.

We identified two common themes for the use of mobile health apps in tertiary breast cancer prevention: clinical care coordination and health related quality of life during and after a breast cancer diagnosis. Cancer care coordination studies focused on the support and communication between the breast cancer patient and the physician (32, 41, 47, 48, 66, 68), as well as specific aspects of cancer care coordination, such as symptomology (12, 14, 23, 27, 52), medication adherence (23, 34, 38, 45, 66), and ambulatory surgery (7, 8). Research using apps designed to improve health related quality of life focused on general lifestyle management (30, 42, 56, 60, 64, 69), weight management (61, 66, 67), depression and breast cancer related distress (12, 17, 21, 23, 37, 63), social support (12, 40, 50, 51), sleep (20), and physical activity during and after a breast cancer diagnosis (9, 11, 22, 24, 25, 28, 29, 33, 35, 36, 46, 55, 59, 65). The use of mobile apps for tertiary cancer prevention was preferred in contrast to usual standard of care practices. For example, multiple studies reported that cancer patients and survivors were willing, and had a preference for, receiving clinical care coordination support (13, 15, 16) and health-related quality of life interventions (53, 62) through apps.

In addition to the two main themes identified, we also found that tertiary prevention apps were used to improve measurement and provide real-time data for assessment and prediction. For example, Timmerman et al. subjectively measured fatigue in 18 cancer survivors by administering the Visual Analog Scale on a smartphone 3 times per day (25). In addition, Langer et al. had cancer patient and spouse dyads systematically record their thoughts via a smart phone twice a day for 14 consecutive days to assess communication (51). Information collected from mobile apps was also validated against other metrics. For instance, Kim et al. found that daily self-reported depression ratings collected by a mobile mental-health application provided comparable results as traditional one-time in-clinic assessment of depression and that higher accuracy of depression was achieved with greater adherence to mobile app use (21). Lastly, information collected via mobile applications was utilized to improve prediction of breast cancer-specific mortality and breast cancer recurrence (31, 57). While risk modeling is a common tool used in clinical practice to inform individuals of their individual cancer risk, Parades-Aracil et al. integrated these risk models into an app making the risk measurement tool more accessible for clinical use.

**Figure 5:**
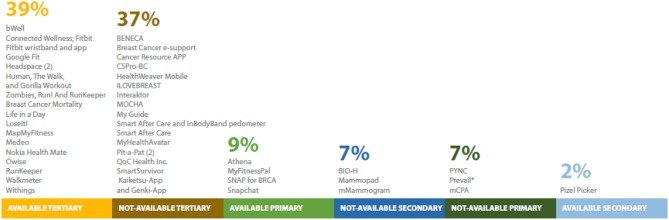
Names and number of publicly-available apps used for breast cancer prevention research (*n* = 69 unique studies). Twenty-one studies excluded because no app name was provided or no app was developed. *Name provided at request of author.

The vast majority of the apps we identified for clinical care coordination were not named in the study or publicly available, but rather developed for each specific study. In contrast, studies using apps to improve health related quality of life were more readily available for public use in the Apple and/or Android app store (Figure 5).

#### Secondary Prevention

We identified 9 studies (8 unique) that used apps for secondary breast cancer prevention in the following phases: development (37.5%), feasibility (25%), pilot (12.5%), and effectiveness (62.5%); with three articles reporting on multiple study phases (see Table 2).

**Table 2:** Articles using mobile apps for secondary breast cancer prevention (*n* = 9 eligible studies).

**References**	**Type of study**	**Population (sample size)**	**Location**	**Outcomes**
Cardos et al. (72)	Feasibility	Community sample of females (16)	Romania	App use and experience
Eden et al. (73)	Pilot + Effectiveness	Clinic sample of females (100)	US	Decisional conflict and intention to screen
Ginsburg et al. (74)	Effectiveness	Women with abnormal clinical breast examination (556)	Bangladesh	Adherence to screening
Heo et al. (19)	Development + Effectiveness	Community sample of females (45)	Korea	Adherence to screening
Jiao et al. (10)	Development	N/A	China	Colorimetric detection of breast cancer cells
Keohane et al. (18)	Effectiveness	Breast clinic sample (84)	Ireland	Knowledge of risk
Lee et al. (75)	Effectiveness + Feasibility	Community sample, Korean American women (120)	US	Knowledge and adherence to screening; app use and experience
Lee et al. (58)^*^	Development	Community sample, Korean American women (14)	US	App interest and preferences
Tewary et al. (76)	Development + Measurement	Breast cancer tissue samples (30)	India	Automated Ki67 proliferation index scoring

* *Duplicate articles are indented*.

*US, United States*.

We identified only one theme in the studies of secondary prevention; with one exception (72), all studies that involved human subjects were effectiveness studies that targeted breast cancer screening behaviors, especially among underserved populations and high-risk women (18, 19, 73–75). For example, Eden et al. found that among rural women aged 40–49 years, apps were effective at reducing decisional conflict and increasing self-efficacy around mammography (73). Two studies used mobile apps to increase breast-screening practices in Korean women. Heo et al. successfully introduced an app to increase breast self-examination among young Korean women (average 29.5 ± 5.9 years) (19). In addition, Lee et al. found that in comparison to the usual care control group that received a printed brochure, Korean American women in the intervention group that received access to a mobile mammography app with health navigator services, showed significantly increased knowledge of breast cancer and greater readiness for mammography (75). Similar to Lee et al., other studies also examined if breast cancer screening is improved when pairing mobile apps with community health navigators (18, 74).

Two developmental studies used apps to innovate breast cancer detection strategies. The SmartIHC-Analyzer mobile app automates scoring of Ki-67 protein, a hallmark for assessing cell proliferation rate during cancer progression (76). The Pixel Picker mobile app rapidly detects breast cancer cells (10).

With one exception (10), none of the mobile apps for secondary prevention were publicly available at the time of this review (Figure 5).

#### Primary Prevention

We identified 10 articles (9 unique) that focused on the use of mobile apps for primary breast cancer prevention (see Table 3). The articles ranged across the following research phases: development (30%), feasibility (20%), protocols (20%), and effectiveness (30%).

**Table 3:** Articles using mobile apps for primary breast cancer prevention (*n* = 10 eligible studies).

**References**	**Type of study**	**Population (sample size)**	**Location**	**Outcomes**
Alanzi et al. (77)	Effectiveness	Community sample of female students (200)	Kingdom of Saudi Arabia	Breast cancer awareness; Guidelines; High-risk;
Businelle et al. (78)	Effectiveness	Hospital sample (92)	US	Smoking lapse; High-risk
Cohen et al. (79)	Feasibility	Community sample of females with BRCA mutation (102)	US	Awareness; Guidelines
Scherr et al. (80)^*^	Development	Community sample of females with BRCA mutation (14) and healthcare providers who work with BRCA carriers (3)	US	App preferences; Framework
Coughlin et al. (81)	Development	Community sample (5)	US	App preferences; Framework; Literacy
Hartman et al. (82)	Effectiveness	Breast clinic sample (54)	US	Weight gain and physical activity; High-risk; Framework
Kratzke et al. (83)	Development	Community sample of female students (546)	US	App preferences; Framework; Self-efficacy
Loef et al. (26)	Protocol	Healthcare workers (1960)	Netherlands	Infection susceptibility; High-risk
Smith et al. (64)	Protocol	Breast cancer survivors, African American (12)	US	App preferences; Guidelines; Framework
Bravo et al. (84)	Feasibility	Breast clinic sample (15)	US	Acceptability and usability; Literacy

* *Duplicate articles are indented*.

*US, United States*.

We identified three common themes for the use of mobile health apps in primary breast cancer prevention: knowledge and adherence to screening guidelines, the targeting of high-risk populations, and the incorporation of theoretical frameworks. Primary prevention studies focused on apps that increased breast cancer prevention knowledge and adherence to breast cancer guidelines and surveillance (77, 79, 80, 83–85). Six of the 9 studies used existing guidelines to inform their apps (77, 80, 81, 83, 85). For example, in designing an app to help women reduce their risk of breast cancer through healthy behaviors, Coughlin et al. (81) included evidence-based information provided by the National Cancer Institute, the Centers for Disease Control and Prevention, and the American Cancer Society. In addition, a protocol study that provided healthy food recipes through the app aimed to assess adherence to diet and physical activity guidelines for cancer survivors set out by the American Institute for Cancer Research (85) and the investigators of an effectiveness study based the content of their app on the Saudi Cancer Foundation guidelines (77). Four studies focused on encouraging healthy behaviors that reduced the risk of breast cancer (78, 81, 82, 85).

The targeted population for these primary prevention studies was primarily women at high risk for breast cancer (77, 79, 80, 82, 83) including post-menopausal women with high Gail risk scores (82), *BRCA* mutation carriers (79, 80), and African American women, who experience greater breast cancer disparities (85). Some studies also targeted broader populations that engaged in behaviors associated with higher breast cancer risk, such as smoking (78) and night shift work (26). In the latter, Loef et al. described the protocol for an observational cohort of health workers in the Netherlands in which an app will be used to collect daily measures of infection to investigate how night shift work impacts health outcomes that are related to carcinogenesis (26). Therefore, apps are used both to increase knowledge about breast cancer risk and prevention in targeted populations (78, 85), as well as to identify new risk factors in high risk populations (26).

Many of the primary prevention studies incorporated theoretical frameworks for behavior change. The development studies incorporated the Common Sense Model of Behavior Theory (81), Health Information Model (83), and the Messaging Model for Health Communication Campaigns framework (80). One protocol study used both the Health Belief Theory and Theory of Planned Behavior Models (64). One effectiveness study based their study design on a Social Cognitive Theory (82). None of the feasibility studies mentioned a theoretical framework.

In addition to the three themes, we found that several key concepts were vital to implementing primary prevention research with apps, including literacy (specific to health and ehealth), self-efficacy (with a distinction between active and passive information seeking), and user-friendly scheduling tools. For example, literacy and self-efficacy were important in a study among college women that applied a family-based life course approach to breast cancer prevention (83). Given college-age women may adopt healthy lifestyles that are important for cancer risk reduction, Kratze et al. found that the app proved useful in knowledge transfer of breast health awareness while also assisting in daughter-initiated communication with their mothers regarding screenings and health information. The need for user-friendly tools, such as scheduling assistants, emerged in a study of guideline adherence among BRCA carriers. Although their awareness of surveillance guidelines was high, adherence was low and half of respondents indicated they had a difficult time remembering to schedule appointments (79). Thus, the app was designed to remind users when to seek care personalized to their own risk factors. The use of apps was particularly helpful in increasing effectiveness of behavioral interventions because they enabled dynamic tailoring in the case of smoking cessation (78) and easier self-monitoring in the case of tracking diet and physical activity (85).

With regard to app availability, 4 studies used publicly-available apps (Figure 5) (77, 79, 82, 84). Other studies used pre-existing apps, including My Fitness Pal (82), Snapchat (77), or incorporated their custom app to be used with FitBit and LoseIt! (81). The studies whose apps were not publicly-available either developed apps for research purposes only (85) or did not mention specific information about their app (26, 83). For one study, the author provided the app name upon contact (78).

## Discussion

This systematic review summarizes the emerging literature for breast cancer prevention research using mobile apps. While we found studies across the cancer control continuum, the majority of studies used mobile apps to target tertiary prevention, particularly clinical care coordination and health-related quality of life for breast cancer survivors, as well as to improve the measurement and assessment of symptoms, behaviors, and risk. Fewer mobile apps were used for secondary and primary prevention where outcomes were related to increasing self-efficacy and screening behaviors and tracking and managing health behaviors. The studies reviewed spanned all phases of research in diverse populations in nearly 20 countries. The use of apps in breast cancer research has been increasing since 2010, a trend that will likely continue. Given the ubiquity of smartphones and global burden of breast cancer, there is potential for mobile apps to impact breast cancer trends across the globe.

### Progress Since Previous Reviews

Previous reviews have explored the use of cancer apps, but were not systematically conducted (86), specific to breast cancer (87), or focused on research (4). That being said, our findings suggest that some of the gaps identified by past reviews have begun to be addressed. In particular, we identified that many of the primary prevention studies were grounded in theoretical frameworks and were tailored to different cultural and literacy levels, key points that were not being addressed previously as identified by Coughlin et al. (86). Similar to Coughlin et al. (86) and Giunti et al. (4), we also found that the majority of breast cancer apps were designed for tertiary prevention. We further observed that in studies of secondary and primary prevention, many apps provide information about guidelines for early detection of breast cancer for women identified as high risk. However, given that early onset breast cancer is increasing even in women without a family history of breast cancer, larger scale prevention interventions should be considered for additional populations that current risk models and screening strategies do not identify. We also found that apps could be adapted for studies across the cancer control continuum given that healthy behaviors recommended for primary and tertiary prevention overlap. Thus, in this rapidly growing field, while some gaps have been addressed, others gaps and implementation opportunities are emerging.

### Research Gaps by Cancer Prevention Types

#### Tertiary Prevention Gaps

Given that breast cancer is the most commonly diagnosed cancer in women globally (88) and there are an estimated 3.5 million breast cancer survivors in the US alone (89), it makes sense that the majority of the apps were focused on clinical care coordination and health related quality of life. The majority of the apps we identified for tertiary breast cancer prevention were patient- or survivor-oriented; therefore, they required adherence from the patient/survivor. While this could place a considerable burden on patients/survivors, the repeat and real-time evidence gleaned can be invaluable for patients/survivors in terms of self-management. Furthermore, a small proportion (16%) of studies using apps for tertiary cancer prevention were effectiveness studies. Given the rising rates of breast cancer incidence in low-middle income countries (90), more studies are needed to show the effectiveness of app use, especially in low resource settings.

#### Secondary Prevention Gaps

While a greater proportion of secondary prevention studies were at the effectiveness stage, we found mixed evidence that apps could modify breast cancer screening behaviors, especially among at-risk populations. Lee et al. showed that a mobile phone-app based intervention, in combination with health navigator services, could effectively improve breast cancer knowledge and readiness for mammography (75). Ginsberg et al. also explored the effectiveness of an app, with or without a health navigator service, to increase Bangladeshi women's adherence to attend a clinic-visit after an abnormal clinical breast examination; however, no significant results were found (74). Similarly, an app in conjunction with genetic clinical counseling did not change women's personal perception of risk (18). Effectiveness studies ought to assess if an app could deliver substantial gains in secondary breast cancer prevention outcomes (e.g., education, screening), alone or in combination with other services. Moreover, given early detection of breast cancer is associated with greater survival rates, effectiveness studies that assess outcomes for the implementation of innovative breast cancer screening/detection apps compared to standard of care, would be of great value. This is especially true for areas where there are barriers to mammography screening and/or timely point-of-care diagnostics.

#### Primary Prevention Gaps

The majority of primary prevention studies were aimed at improving the transfer of knowledge and adherence to existing cancer prevention guidelines among women at high risk for breast cancer; however, less research has been conducted with populations at average risk, or on modifiable risk factors to prevent breast cancer. Targeted prevention to high-risk populations is logical given that with limited resources and competing disease risk, resources should be allocated to those who will benefit most. However, if maintaining healthy weight, diet and physical activity can reduce cancer incidence by 26% (91), then apps can help promote sustainable, scalable behavioral change that reduces the risk for many additional chronic diseases (e.g., heart disease, diabetes) for women at average risk as well.

### Global Implementation Implications

As of early 2019, there were over 5.1 billion mobile phone subscribers and this number is growing given the average annual percent increase of 2.9% (92). One could argue that the adoption of smartphone use is faster than the rate of an epidemic. With smartphones, individuals are readily, in real time, self-monitoring health behaviors. And leveraging this self-tracking for the implementation of breast cancer prevention is at our fingertips. Our review suggests that the use of apps for breast cancer prevention is far-reaching. The global rise in incidence rates of breast cancer coupled with a rapid uptake of mobile platforms creates unique prevention opportunities. That being said, it is unclear if the use of apps for breast cancer prevention will mitigate or create greater gaps in health disparities (93). While low to middle income countries have experienced rapid uptake of mobile platforms (94), in these emerging markets, the young, well-educated and higher-income individuals are more likely to use these mobile platforms (93). Thus, an unintended consequence is the creation of breast cancer health disparities in low resource settings; especially for secondary and tertiary prevention. But, thoughtful app developments and implementation of mHealth tools could lead to more inclusive rather than marginalized research (93).

### Opportunities and Recommendations of Mobile App Use Across the Cancer Control Continuum

Given our review, we highlight the following opportunities and/or recommendations with regard to the use of apps across the breast cancer control continuum:

Research is needed to understand the effectiveness of mobile apps for breast cancer primary prevention in women at average risk, but especially in young women. The incidence of invasive breast cancer in young women (age 25–39 years) has risen in the US with an annual percent change of 2.7% for white non-Hispanic women and 3.1% for black non-Hispanic women from 1976 to 2009 (1). Moreover, while global incidence rates for young women under 50 years are similar, independent of country-level income, mortality rates are higher in women in low-middle income and low-income countries (95). Many behavioral risk factors for breast cancer are modifiable, so the potential impact of app technology for breast cancer prevention in young women is particularly powerful given that this age group has come of age with apps and they do not need to be taught or convinced of their usefulness (93).

Breast cancer apps should be readily available. Only about half of the apps in our review were publicly available in the Apple and/or Android app store. The majority of apps readily available for public use were health related apps; whereas, apps catering to secondary prevention (breast cancer screening/detection) and tertiary prevention (continuing cancer care) were not readily available. Even for primary prevention, Cohen et al. found that over 200 potential users from 68 countries outside of the US tried to access the SNAP for BRCA app, but potential users could not download the app as it required a study code (79). Without making developed apps readily available and usable, there is limited possibility of updating, adapting, validating, disseminating, or further testing the app for effectiveness in diverse populations and settings. Researchers should also take advantage of already available apps, especially popular ones (e.g., Fitbit, Headspace), as there is less upfront person time and financial expenses compared to *de novo* app development. Popular apps carry the benefit of having a strong infrastructure given that software is routinely updated, designs are improved, and new features are added (82). However, an inherent limitation of readily available apps is that the speed of the research does not often advance at the speed of mobile app technology; therefore, researchers have limited control over app developments and the changes that may directly or indirectly impact the study.

Researchers should capitalize on the opportunity apps provide to collect information on exposures and outcomes of interest that have traditionally been difficult to measure. Not only does mobile app technology allow researchers to obtain repeat real-time data, mobile data measurement and collection reduces in-person study staff assistance, while not fully replacing study staff. Study staff will likely remain essential, especially for study implementation in low-middle income and hard to reach populations (84).

### Limitations

This review is not without limitations. First, the advent of mobile apps is relatively recent and research in this area is rapidly changing. As a result, articles may have been missed that were not indexed with the search terms selected. To counteract this possibility, we broadened our search to include the full-text rather than just MeSH or keywords. Second, our review may also be missing studies that addressed breast cancer risk factors, such as obesity, but do not make an explicit reference to breast cancer. This likely deflated the number of articles identified as primary prevention; however, a more exhaustive review of all mobile apps being used for breast cancer risk factors was beyond the scope of this study. Finally, we included two databases in our search strategy, so gray literature and clinical trials with unpublished findings were not included.

### Conclusions

The use of mobile apps for breast cancer prevention research is rapidly growing. Our systematic review suggests that while some gaps identified in previous reviews have already been addressed, new challenges have emerged. For mobile app interventions to have a global impact across the cancer control continuum, researchers will need to continue to invest in primary and secondary prevention research studies, as well as studies that are farther along in the research phase, in order to demonstrate the potential impact on outcomes relevant to breast cancer.

## Author Contributions

LH and JM conceptualized the study and all authors (LH, RH, JM) formulated the study design. RH managed the literature search and reviewed all articles and LH and JM independently reviewed a subset of articles. All authors drafted the initial manuscript, reviewed and revised the final manuscript for critical and important intellectual content, approved the final manuscript, and agree to be accountable for all aspects of the work.

### Conflict of Interest

The authors declare that the research was conducted in the absence of any commercial or financial relationships that could be construed as a potential conflict of interest.
