# Supplementary material for: Mobilizing Breast Cancer Prevention Research Through Smartphone Apps: A Systematic Review of the Literature
Source: Front Public Health. 2019 Nov 6;7:298. doi: 10.3389/fpubh.2019.00298 (PMC6851054; doi:10.3389/fpubh.2019.00298)
Supplement: Supplementary file 1 [file Table_1.docx]

**Supplementary table 1**

("Breast Neoplasms"[Mesh] OR ("breast cancer" or "breast neoplasms") OR (("cancer" or "cancers") AND "breast")) AND ("Mobile Applications"[Mesh] OR "smartphone" OR “smartphones” OR "smart phone" OR “smart phones” OR "mobile app" OR “mobile apps” OR "mobile application” OR “mobile applications" OR “mobile phone app” OR "mobile phone apps" OR “mobile phone application” OR “mobile phone applications” OR “phone app”)
